# FDA endorses NALIRIFOX for metastatic pancreatic adenocarcinoma: an editorial

**DOI:** 10.1097/MS9.0000000000002564

**Published:** 2024-09-10

**Authors:** Wajiha Urooj, Bisma Ahmed, Yumna Shahzad, Amal Siddiqui, Zaib Un Nisa Mughal, Khabab Abbasher Hussien Mohamed Ahmed

**Affiliations:** a Department of Medicine, Jinnah Sindh Medical University, Karachi, Pakistan; b Faculty of Medicine, University of Khartoum, Khartoum, Sudan

## Background

Pancreatic cancer incidence has been increasing over the past years as it now ranks the seventh most prevalent malignant tumour with 466 003 cases globally. Pancreatic cancer has a poor prognosis, with a 5-year survival rate of less than 9%, primarily because of nonspecific symptoms and late detection. In 2020, pancreatic cancer caused 132 134 deaths across Europe, with 140 116 new cases reported annually. Consequently, pancreatic cancer accounts for nearly 29% of all cancer-related deaths in Europe. Pancreatic ductal adenocarcinoma (PDAC) is the most common and fatal exocrine tumour, accounting for over 85% of cases and the highest mortality rate among pancreatic neoplasms^1^.

Approximately two-thirds of PDAC develop in the head of the pancreas, while the remaining cases are equally distributed between the body and tail of the gland^2^. Clinical features are commonly nonspecific including back pain, dyspepsia, nausea and weight loss making its early diagnosis difficult^1,2^.

Hereditary factors contribute to 10% of pancreatic cancer cases. The risk increases 9-fold if one first-degree relative has been diagnosed and rises to 32-fold if at least three first-degree relatives have the disease. Non-modifiable, non-genetic risk factors for pancreatic cancer include age and sex. In 2020, the global incidence of pancreatic cancer was higher in men (5.7 per 100 000) compared to women (4.1 per 100 000). The incidence of pancreatic cancer rises with age, with over 90% of patients being over 50 years old. The median age at diagnosis ranges from 70 to 80 years^1^. Identified modifiable risk factors for pancreatic cancer include smoking, obesity, low physical activity, type 2 diabetes, and alcohol consumption. Studies indicate that obesity is particularly linked to PDAC^1–3^.

PDAC incidence is highest among black individuals in the USA, with higher risk among chronic pancreatitis patients and those with previously resected PDAC. PDAC are whitish masses with irregular borders, often infiltrating surrounding structures like peri-pancreatic adipose tissue, the duodenal wall, distal choledocus, and ampulla of Vater. Precursors of PDAC include pancreatic intraepithelial neoplasm (PanIN), intraductal papillary mucinous neoplasm (IPMN), intraductal oncocytic papillary neoplasms (IOPN), intraductal tubulopapillary neoplasm (ITPN), and mucinous cystic neoplasm (MCN). All these lesions can progress into the invasive counterpart, which is represented by PDAC^2^.

## Treatment options for metastatic pancreatic adenocarcinoma

Treatment options depend on tumour staging and include surgical resection, adjuvant or neoadjuvant chemotherapy or radiochemotherapy for an advanced disease that is no longer resectable^1^. First-line treatment options for metastatic PDAC include gemcitabine/nab-paclitaxel and FOLFIRINOX. Both treatments have demonstrated better survival outcomes compared to gemcitabine monotherapy, although overall survival rates remain low^4^.

## NALIRIFOX

On 13 February 2024, NALIRIFOX received FDA approval. NALIRIFOX, the newly FDA-approved frontline treatment for patients with metastatic pancreatic adenocarcinoma, is the chemotherapy regimen comprises a combination of liposomal irinotecan (Onivyde) with oxaliplatin, 5-fluorouracil, and leucovorin^5^.

NALIRIFOX is administered biweekly with the following regimen: a 90-min intravenous infusion of 50 mg/m² of irinotecan liposome, a 120-min intravenous infusion of 60 mg/m² of oxaliplatin, a 30-min intravenous infusion of 400 mg/m² of leucovorin, and a 46-h intravenous infusion of 2400 mg/m² of fluorouracil^5,6^.

The former chemotherapy combination, FOLFIRINOX, can cause significant adverse effects. In order to enhance the efficacy and minimise side effects of FOLFIRINOX, the study investigates the viability and safety of replacing non-liposomal irinotecan with liposomal irinotecan (nal-IRI) in order to enhance tumour medication distribution and perhaps lower toxicity. The NALIRIFOX regimen may prove to be safer and more efficient when used in the preoperative phase^7^.

NAL-IRI is a liposomal form of the topoisomerase I inhibitor, irinotecan. By encapsulating irinotecan within liposomes, NAL-IRI prevents its premature conversion into the active metabolite SN-38 in the liver, thus prolonging its presence in the body and enhancing its accumulation within tumour tissues. Once deposited, phagocytic cells convert NAL-IRI into SN-38, increasing its potency by 100–1000 times^8^.

## Clinical trials and efficacy

The phase I/II study, conducted in an open-label, two-part design, recruited participants from 26 October 2015, to 29 October 2018. Spanning across 21 sites located in Australia, Spain, and the USA, patients underwent treatment every 2 weeks, occurring on days 1 and 15 of each 28-day cycle^4^.

The nITRO trial, a phase 2 study conducted in Italy, aimed to identify individuals aged 18 years or older with surgically resectable pancreatic ductal adenocarcinoma. Over the course of the trial, which ran from May 2018 to May 2022, 107 patients were enroled. These patients initiated preoperative treatment, resulting in a disease-control rate of 92.9%. Out of the total, 87 patients underwent surgical exploration, while 11 were found to have metastatic disease, and unfortunately, 1 patient passed away. The R0 resection rate, indicating complete tumour removal, stood at 65.3%. Following surgery, 49 patients completed three cycles of postoperative treatment. The median overall survival was recorded at 32.3 months, with disease-free survival and surgery-related survival measured at 19.3 and 40.3 months, respectively^9^.

The multinational NAPOLI 3 trial, a randomized, open-label, phase 3 study, was conducted at 187 community and academic centres across 18 countries globally, including Europe, North America, South America, Asia, and Australia. In this study^9^, 82% White, 5% Asian, 3% Black or African American and 2% other races receive NALIRIFOX. Results from this trial demonstrated significant enhancements in overall survival and progression-free survival among patients with metastatic pancreatic ductal adenocarcinoma treated with the NALIRIFOX regimen compared to those receiving nab-paclitaxel and gemcitabine^9^.

Between 19 February 2020, and 17 August 2021, a total of 770 patients were randomly assigned to either receive NALIRIFOX (383 patients) or nab-paclitaxel and gemcitabine (387 patients). The safety analysis included 749 patients (NALIRIFOX: 370 patients; nab-paclitaxel and gemcitabine: 379 patients) who had received at least one dose of the study medication. As of the data cutoff date, 44 patients (12%) in the NALIRIFOX group and 7 patients (2%) in the nab-paclitaxel and gemcitabine group were still undergoing treatment. The primary reason for discontinuation was disease progression, reported in 184 patients (48%) in the NALIRIFOX group and 177 patients (46%) in the nab-paclitaxel and gemcitabine group^10^.

An analysis of seven trials involving 2581 patients revealed that individuals treated with NALIRIFOX (383 patients) or FOLFIRINOX (433 patients) experienced longer median progression-free survival (PFS) compared to those treated with GEM-NABP (1756 patients). Moreover, GEM-NABP was associated with poorer overall survival (OS) in contrast to NALIRIFOX, while no significant difference was noted between FOLFIRINOX and NALIRIFOX. Additionally, there were no statistically significant variances in overall response rates (ORR) among patients receiving NALIRIFOX (41.8%), FOLFIRINOX (31.6%), and GEM-NABP (35.0%)^10^.

Comparing NALIRIFOX to previous regimens, there is a considerable increase in the prevalence of severe diarrhoea, but a decrease in the incidence of peripheral neuropathy and severe haematological toxic effects. The fact that NALIRIFOX contains a lower dose of oxaliplatin than FOLFIRINOX could account for the decreased prevalence of peripheral neuropathy. Its moderate toxicity profile and high overall response rate make it an intriguing regimen in some contexts, such as neoadjuvant or perioperative therapy, where optimising tumour shrinking and minimising adverse effects are the main goals^6^.

NALIRIFOX treatment outperformed gemcitabine plus nab-paclitaxel in all patient subgroups, particularly those with high-performance status (ECOG 0) and multiple metastatic sites. Age also had an important influence, with individuals over 65 receiving a greater advantage. This effect was also shown in the PRODIGE study, where FOLFIRINOX demonstrated better benefits in older patients than in younger ones.

## Safety and side effects

The NALIRIFOX group had a reduced percentage of grade 3–4 peripheral neuropathy compared to the nab-paclitaxel and gemcitabine groups (3·2% vs. 5·8, while also exhibited decreased incidence of haematological grade 3–4 treatment-emergent side effects, such as neutropenia, anaemia, and thrombocytopenia, when compared to the nab-paclitaxel plus gemcitabine group. The safety profile of NALIRIFOX may be influenced by factors such as reducing oxaliplatin dosages to decrease toxicities in cumulative peripheral neuropathy cases and using irinotecan’s liposomal formulation to enhance tumour exposure while reducing systemic toxicity^6^.

The commonly reported side effects of NALIRIFOX include diarrhoea, fatigue, nausea, vomiting, reduced appetite, abdominal pain, mucosal inflammation, constipation, and weight loss. Laboratory investigations frequently revealed abnormalities such as decreased neutrophil counts, reduced potassium levels, diminished lymphocytes, and decreased haemoglobin levels^5^.

The cost-effectiveness of the NALIRIFOX regimen will be evaluated through further research, with final medication pricing negotiated with national reimbursement organisations. NALIRIFOX had a favourable toxicity profile, with lower G3–4 neutropenia rates (14.1%) than FOLFIRINOX (45.7%). However, patients who received FOLFIRINOX with all components had the greatest rate of adverse events and total cost per administration cycle. A US study found that Medicare patients receiving second- or third-line liposomal irinotecan had lower costs than patients who received all other regimens, due to lower inpatient admission patterns, compared to other regimens^6^.

## Conclusion

In conclusion, the FDA’s approval of NALIRIFOX for metastatic pancreatic adenocarcinoma signals a significant leap forward in the quest for effective treatments. This milestone not only offers newfound hope for patients but also underscores the importance of ongoing innovation in cancer care. As we embrace this breakthrough, it’s crucial to remain vigilant in monitoring its impact on patient outcomes and to explore avenues for further refinement. With NALIRIFOX, we embark on a promising journey toward bettering the lives of those affected by this challenging disease.

## Ethical approval

Not applicable.

## Consent

Not applicable.

## Source of funding

This study received no funding or financial support.

## Author contribution

B.A. and Z.U.N.M. did the conceptualization. B.A., W.U., A.S., Z.U.N.M., K.A.H.M.A. and Y.S. conducted the literature and drafting of the manuscript. Z.U.N.M. and injunction performed the editing and supervision. All authors have read and agreed to the final version of the manuscript.

## Conflicts of interest disclosure

The authors declare no conflicts of interest.

## Research registration unique identifying number (UIN)

1. 1 Name of the registry: Not required because this is an editorial.
2. 2 Unique Identifying number or registration ID: Not applicable because this is an editorial.
3. 3 Hyperlink to your specific registration (must be publicly accessible and will be checked).

## Guarantor

Khabab Abbasher Hussien Mohamed Ahmed.

## Data availability statement

Not applicable.

## Provenance and peer review

Not commissioned, externally peer-reviewed.

## References

[1] Partyka O Pajewska M Kwaśniewska D. Overview of pancreatic cancer epidemiology in Europe and recommendations for screening in high-risk populations. Cancers 2023;15:3634. 37509296 10.3390/cancers15143634 PMC10377815

[2] Luchini C Grillo F Fassan M. Malignant epithelial/exocrine tumors of the pancreas. Pathologica 2020;112:210. 10.32074/1591-951X-167 PMC7931574 33179623

[3] Cai J Lu B Chen H. The impacts of exposure to risk factors during youth on the increasing global trend of early-onset pancreatic cancer. Public Health 2024;229:65–72. 38402665 10.1016/j.puhe.2023.11.006

[4] Wainberg ZA Bekaii-Saab T Boland PM. First-line liposomal irinotecan with oxaliplatin, 5-fluorouracil and leucovorin (NALIRIFOX) in pancreatic ductal adenocarcinoma: a phase I/II study. Eur J Cancer 2021;151:14–24. 33957442 10.1016/j.ejca.2021.03.028

[5] Trivano M Ipsen IPN Singhal R. A phase II study of neoadjuvant liposomal irinotecan with 5-FU and oxaliplatin (NALIRIFOX) in pancreatic adenocarcinoma. Future Oncol 2023;19:1843–1853. 10.2217/fon-2023-0256 PMC10594143 37753702

[6] Melisi D Casalino S Pietrobono S. Integration of liposomal irinotecan in the first-line treatment of metastatic pancreatic cancer: try to do not think about the white bear. Therap Adv Med Oncol 2024;16:17588359241234487. 38584763 10.1177/17588359241234487 PMC10996353

[7] Hubner RA Cubillo A Blanc JF. Quality of life in metastatic pancreatic cancer patients receiving liposomal irinotecan plus 5-fluorouracil and leucovorin. Eur J Cancer 2019;106:24–33. 30458340 10.1016/j.ejca.2018.09.029

[8] Melisi D Zecchetto C Merz V. Perioperative NALIRIFOX in patients with resectable pancreatic ductal adenocarcinoma: the open-label, multicenter, phase II nITRO trial. Eur J Cancer 2024;196:113430. 37995598 10.1016/j.ejca.2023.113430

[9] Wainberg ZA Melisi D Macarulla T. NALIRIFOX versus nab-paclitaxel and gemcitabine in treatment-naive patients with metastatic pancreatic ductal adenocarcinoma (NAPOLI 3): a randomised, open-label, phase 3 trial. Lancet 2023;402:1272–1281. 37708904 10.1016/S0140-6736(23)01366-1 PMC11664154

[10] Nichetti F Rota S Ambrosini P. NALIRIFOX, FOLFIRINOX, and gemcitabine with Nab-Paclitaxel as first-line chemotherapy for metastatic pancreatic cancer: a systematic review and meta-analysis. JAMA Netw Open 2024;7:e2350756. 38190183 10.1001/jamanetworkopen.2023.50756 PMC10774994

